# Travel despite the COVID-19 pandemic: Implications for tourism recovery

**DOI:** 10.3389/fpsyg.2022.1015421

**Published:** 2022-10-05

**Authors:** Hongbo Liu, Bingjie Liu-Lastres, Li Zeng, Holly Donohoe

**Affiliations:** ^1^School of Hospitality and Tourism Management, University of Surrey, Surrey, United Kingdom; ^2^Department of Tourism, Event and Sport Management, Indiana University-Purdue University Indianapolis, Indianapolis, IN, United States; ^3^College of History, Culture and Tourism, Jiangxi Normal University, Nanchang, China; ^4^Flagler College, Saint Augustine, Florida, FL, United States

**Keywords:** COVID-19, traveling during pandemic, health belief model, mixed-methods, government trust, psychological capital, past travel experience

## Abstract

The COVID-19 pandemic has devastated the global tourism industry. This study explores why some Chinese residents travel during the pandemic. A mixed-methods research design was adopted, guided by the health belief model and relevant literature. Through 21 interviews with Chinese tourists who took an overnight leisure trip in May 2020, and a national survey among Chinese residents, this study explored factors influencing Chinese residents’ travel-related decisions and behaviors during the pandemic. Results outline the influences of health beliefs, government trust, past travel experience, and psychological capital on tourists’ risk-reduction behaviors. Theoretical and practical implications are provided regarding tourism recovery during pandemics.

## Introduction

Coronavirus or COVID-19, an infectious disease caused by the SARS-CoV-2 virus, was first identified in Wuhan, China, and soon disseminated beyond China’s borders due to its highly contagious nature (McKibbin and Fernando, 2021). This health emergency swiftly escalated, and the World Health Organization (WHO) declared COVID-19 a global pandemic in March 2020. In 2022 and now, 2 years since COVID-19 was first observed in China, it continues to maintain its “global pandemic” status, and this is not expected to change as new variants such as Delta and Omicron emerge (WHO, 2022). In addition to the health impacts, the global pandemic has had unprecedented economic impacts on local-to-global scales (McKibbin and Fernando, 2021). Research confirms that the pandemic led to a severe global recession, with the economic costs of the pandemic in the hundreds of trillions of dollars (World Bank, 2020). This marks not only the worst economic recession since the Great Depression but also a “severe setback” with long-lasting impacts effects in both developed and developing countries, with the fault lines between them widening (World Bank, 2020; UNWTO, 2022a).

The tourism economy was among the first to experience the shock and devastating effects of COVID-19 and the strategic response to contain the outbreak. Widespread quarantines and travel bans resulted in cancelled flights and cruises and closings of resorts, attractions, restaurants, and tourism attractions and gathering places (Gössling et al., 2020). These restrictions sometimes lead to the perception that avoiding travel can be a way to protect people from the pandemic (Bae and Chang, 2021). However, reducing social contacts only represents one aspect of the non-pharmacological interventions, and one’s risk reduction behaviors may assume a broader scope than avoiding travel. International travel essentially came to a screeching halt by April of 2020, and despite a rollback of some restrictions, international tourist arrivals declined by 74 percent in 2020 and by 71 percent in 2021 compared to the pre-pandemic records in 2019 (UNWTO, 2022b). Uneven vaccination rates across the globe and new COVID-19 strains are contributing to an unpredictable situation that is expected to impact the tourism sector’s already slow and fragile recovery (Mickensey & Company, 2022).

It is possible to infer from research concerned with people’s post-COVID/post-pandemic travel intentions that the pandemic will continue to slow tourism’s recovery long after the pandemic has receded. In his book, this phenomenon was observed during and after the SARS epidemic in 2003 and aptly named the “China Syndrome” by author Karl Greenfield (Greenfeld, 2009). Therein, he describes the true extent of the SARS epidemic in terms of its long-term effects on all the SARS-hit areas and surrounding regions, such as economic damages, psychological impact, travel restrictions, and diminished international travels (Wilder-Smith, 2006; Greenfeld, 2009). Greenfeld (2009) predicted that the “China Syndrome” would presage other pandemics to come, and the current pandemic is proving this to be true. Despite China’s much-improved response to COVID-19 over SARS, the associated health concerns are tempering people’s willingness to travel, and this is particularly evident in destinations where risk is perceived to be heightened by the government’s response (Zheng et al., 2021). The problem, as United Nations Secretary-General Antonio Guterres stresses, is that tourism’s role as ‘one of the most important economic sectors, providing livelihoods to hundreds of millions of people while boosting economies and enabling countries to thrive,’ has been significantly impacted by COVID-19 and these impacts will be felt for decades to come (UNWTO, 2020).

As the source of the outbreak, China has been portrayed by the media as the epicenter of infection (Knight et al., 2020; Lu and Atadil, 2021). While global travel ground to a halt in 2020, domestic travel within China in the same year generated nearly 104 million trips during the May Day holiday (News Xinhua, 2020) and more than 637 million people travelled during the Golden Week holiday in October, generating approximately 466 billion yuan (~US$68.6 billion) in tourist income (Pitreli, 2020). Despite the pandemics’ clear health risks, Chinese people traveled, and this suggests that health beliefs and other potential factors may have been driving domestic travel despite the health crisis.

China’s strong recovery is partly due to the government’s rapid response, including nonpharmaceutical policies intended to slow viral transmission (Zheng et al., 2021). Aside from governmental efforts, potential tourists’ responses to risk vary: some may avoid travelling altogether while others engage in self-protective measures or take trips as usual (Sönmez and Graefe, 1998; Zheng et al., 2021, 2022). Tourists’ behavioral responses to the pandemic depend on their psychological states and past travel experiences, which could shape their attitudes and perceptions about the pandemic. Although several researchers (Bae and Chang, 2021; Liu-Lastres et al., 2021; Neuburger and Egger, 2021; Pappas, 2021; Sánchez-Cañizares et al., 2021; Zheng et al., 2021, 2022) have explored people’s post-pandemic travel plans, few have examined why people are willing to take trips and their risk reduction behaviors during travel amidst the pandemic. This understanding is critical to destination recovery; such information can clarify potential tourists’ needs, wants, and preferences during the recovery period (Gursoy and Chi, 2020).

As a response to this research need, the purpose of this study is to identify what factors influence travelers’ decision-making and risk reduction behaviors during the COVID-19 pandemic. More specifically, this research was guided by the question “Why do Chinese residents travel during the COVID-19 pandemic?” The Health Belief Model (HBM) has been established as a valid theoretical framework to understand the relationships between infectious diseases, health beliefs and health behaviors (Donohoe et al., 2018; Naseer et al., 2021; Suess et al., 2022). Thus, we hypothesized that the HBM might have explanatory relevance for travel behavior during the COVID-19 pandemic and utilized HBM as the theoretical foundation. Guided by the health belief model and related literature, this study adopted a mixed-methods design, including 21 semi-structured interviews and a national survey of 901 Chinese urban residents. Results delineate the factors shaping tourists’ decisions to travel during a global pandemic.

## Literature review

### An overview of COVID-19 related studies

In an attempt to predict and understand the short and long-term effects of the pandemic on the tourism sector, a growing body of research is evolving. Scholars have built models to forecast the tourism industry’s recovery from the pandemic (Fotiadis et al., 2021; McKibbin and Fernando, 2021; Zhang et al., 2021). Research has also specifically examined individuals’ travel intentions (Liu-Lastres et al., 2021; Neuburger and Egger, 2021), travel avoidance (Zheng et al., 2021), and post-pandemic tourism consumption patterns (Wen et al., 2020). Furthermore, studies have investigated factors underlying COVID-19’s effects on tourists, including health beliefs (Suess et al., 2022), risk perceptions and efficacy beliefs (Liu-Lastres et al., 2021; Zheng et al., 2021), destination image (Lu and Atadil, 2021), trust (Zheng et al., 2022), and pandemic-associated emotions such as fear (Zheng et al., 2021), and anxiety (Liu-Lastres et al., 2021).

Notably, these studies have primarily focused on the influence of COVID-19 on people’s post-pandemic travel intentions, thereby suggesting a re-emergence of a “China Syndrome” but on a now-global scale (Bae and Chang, 2021; Liu-Lastres et al., 2021; Neuburger and Egger, 2021; Zheng et al., 2021, 2022). While pre-COVID research confirms that travelers’ risk perceptions are multidimensional, with health-related risks constituting the main category, their behavioral intentions do not necessarily parallel their actions (Chandon et al., 2005).

To our knowledge, the research has yet to provide insight into why people travel amid an active infectious disease health crisis such as a pandemic. In light of the knowledge that the SARS epidemic and the COVID-19 pandemic were both facilitated by human mobility (Wilder-Smith, 2006; Fotiadis et al., 2021; Mickensey & Company, 2022), understanding the factors that affect a traveler’s health beliefs and actions during a pandemic is crucial for informing travel restrictions and other strategic responses for the management of future infectious disease health crises (Donohoe et al., 2018). It is equally important to understand what affects travelers’ actions during a pandemic and post-pandemic travel as it may provide important insight for the tourism sector and its development of strategies to recover international tourist arrivals in the years to come. Concomitantly, it may be valuable for informing the development of strategic and/or resiliency plans for future health crises (Gursoy and Chi, 2020).

### Conceptual background: Health belief model

HBM is a theoretical model developed in public health research to explain and forecast health-related behaviors (Janz and Becker, 1984). This model posits that individuals’ disease prevention strategies can be predicted by their health beliefs and associated risk perceptions. According to HBM, individuals’ adoption of preventive behavior can be predicted by (a) their perceived susceptibility to and severity of a health risk; (b) the perceived benefits and barriers of taking preventive measures; and (c) their self-efficacy in dealing with this risk (Champion and Skinner, 2008).

Traditionally, HBM has been used to explain various health-related behaviors, and the theory was recently extended to tourism contexts. For instance, it has been applied to study health risk–related preventive behavior among tourists visiting high-altitude destinations (Huang et al., 2020), along with tourists’ intentions to participate in medical tourism (Chaulagain et al., 2020) and forest therapy tourism (Zhao and An, 2021). Given its relationship with public health, HBM has also been adopted to explain people’s tourism- and hospitality-related behavior during the COVID-19 pandemic. Topics of interest include travelers’ intentions to participate in untact tourism (i.e., minimize contact between people during travel; Bae and Chang, 2021) and consumers’ dining behavior (Yang et al., 2020). Although guided by HBM, the two aforementioned studies neither empirically measured nor tested key variables’ influences in light of this theory.

In the event of a major health crisis such as COVID-19, people’s general health-related behavior often revolves around preventive measures (e.g., social distancing and mask-wearing), and perceptions related to the overall situation. HBM hence serves as a logical theoretical foundation to uncover the drivers behind tourists’ behavior during the pandemic. Therefore, empirically exploring the correlations between infection and mobility in terms of COVID-19 and how individuals decide to embark on trips or avoid traveling is particularly essential in furthering our understanding in this area.

### Influences of government trust and psychological capital

The COVID-19 pandemic is relatively unique in that national governments are highly involved in its management. Governments’ crisis intervention and communication efforts have been shown to affect individuals’ perceived risks along with their judgment and understanding of the situation (Slovic et al., 2005). These outcomes can be reflected in the government trust, which encompasses the public’s confidence in governmental measures’ ability to effectively address public health crises and the credibility of provided crisis-related information (van der Weerd et al., 2011). Public health research has empirically demonstrated that trust in government informs the public’s risk perceptions, thereby shaping people’s preventive behavior during health crises (van der Weerd et al., 2011; Ye and Lyu, 2020).

Findings on the influences of government trust on tourists’ decisions during the COVID-19 pandemic appear inconclusive. For example, Fong et al. (2020) reported that perceived government performance in dealing with COVID-19 could enhance tourists’ self-efficacy and anticipated tourism recovery. In contrast, Zheng et al. (2022) observed that trust in government could increase travel fear, ultimately leading to travel avoidance after COVID-19. Wong and Jensen (2020) further argued that trust in government is a double-edged sword: it may encourage preventive behavior through the perceived severity of COVID-19 but discourage preventive behavior through perceived government competence in controlling the outbreak, resulting in underestimated risk. Therefore, more empirical studies are needed to understand the complex roles of trust in government in tourists’ health beliefs and risk perceptions, which could inform tourists’ health-related preventive behavior during the COVID-19 pandemic.

At an individual level, psychological capital is another element that defines one’s attitude and decisions in uncertain times. Originating from positive psychology, the concept of psychological capital captures the positivity in individuals’ psychological states (Luthans et al., 2007b). It is characterized by the dimensions of self-efficacy, hope, optimism, and resilience, all of which play essential roles in individuals’ ability to cope with stressful or threatening situations (Tugade and Fredrickson, 2004; Karademas, 2006; Wang et al., 2019; Zheng et al., 2021). Psychological capital is particularly relevant to COVID-19, a public health crisis that threatens travelers, yet few studies have considered psychological capital’s role in tourists’ responses during pandemic times.

## Research design overview

This study adopts an exploratory sequential mixed-method design for identifying factors influencing tourists’ decisions to travel during a pandemic (Creswell et al., 2003). Mixed-methods research involves combining both qualitative and quantitative components in the same study so as to facilitate a dialogue between them (Ivankova et al., 2006). This study followed an exploratory sequential design, which combines qualitative and quantitative approaches in a sequence of phases whereby the first phase informs the second (Creswell et al., 2003). Such a design is most often used to develop theory and identify the theoretical constructs or variables to be included in a quantitative research instrument or where the objective is to test or refine an instrument to test a hypothesis (Ivankova et al., 2006). The rational for this approach lies in first exploring the topic before determining what variables need to be measured.

**Figure 1:**
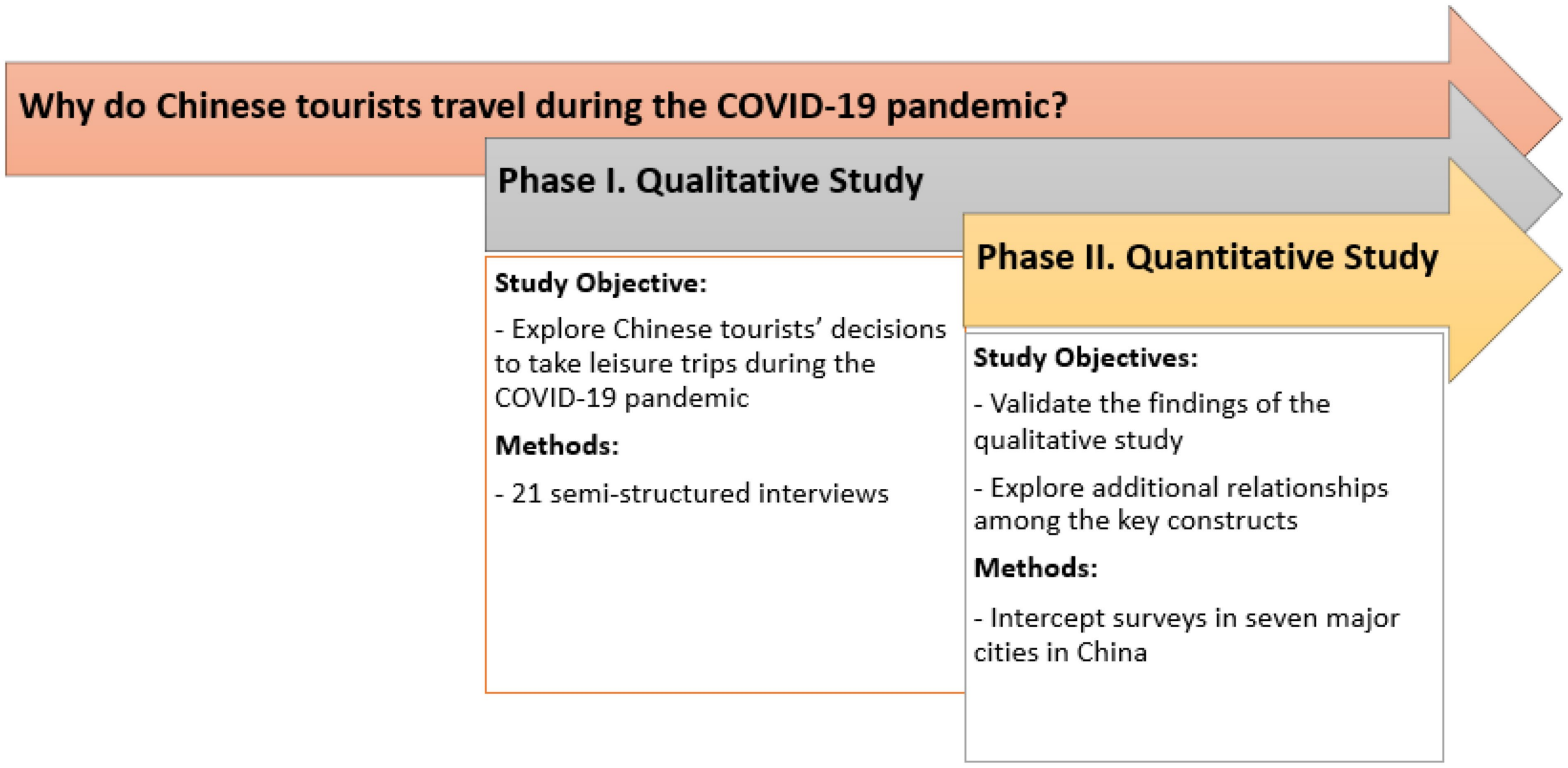
Research design.

As illustrated in Figure 1, this research consisted of two phases. The first phase featured a qualitative approach entailing semi-structured interviews with 21 Chinese tourists who took an overnight leisure trip during the May Day Holiday in early May 2020. Interviews were intended to uncover major factors influencing tourists’ decisions and risk reduction behavior. The second study phase was quantitative and involved intercept surveys in seven major cities in China. The instrument was developed based on the interview findings and related literature. Combining qualitative and quantitative approaches provides a rich understanding of a topic and can unveil the dynamics of social phenomena (Ivankova et al., 2006).

## Qualitative study

### Qualitative research design

The qualitative phase aimed to explore why Chinese tourists would travel during a global pandemic, even though their trips occurred shortly after domestic travel restrictions were lifted. The target population consisted of Chinese tourists who travelled during the 2020 May Day Holiday. Data were collected *via* phone interviews and site interviews. Participants were identified through purposive sampling. Specifically, snowball sampling was employed to recruit participants for phone interviews; the research team initiated this process through their personal networks. The initial participants of phone interviews were identified and recruited from the researchers’ network: individuals who posted their trips taken during the May Day Holiday on social media were invited to take part in the study. They were also asked to recommend other qualified participants upon the interview completion. Regarding site interviews, three research assistants were trained and dispatched to the three most popular tourist attractions in Jiangxi Province to conduct interviews. Participants were identified through street interception. To be eligible to participate, all interview participants were required to be over 18 years old and to have taken at least one overnight, cross-city leisure trip during the May Day Holiday. Three screening questions were used to identify qualified participants: (1) what is your age? (2) where do you come from? (3) how many nights did you spend/do you plan to stay at your destination? A total of six phone interviews and 15 site interviews were conducted.

All interviews were semi-structured and based on the same protocol: “Why did you decide to travel during the pandemic?” (2) “How did you feel about your trip? Did you feel safe and why?” (3) “Did you do anything to protect yourself against COVID-19 during your trip? If so, what measures did you take?” (4) “Do you travel a lot? How often did you travel last year?” The interview questions were developed around the research question in this study: “Why do Chinese residents travel during the COVID-19 pandemic?” All interviews were audio-recorded and transcribed verbatim by the research team with participants’ consent. Interviews were between 15 and 35 minutes long. The data were analyzed following the process outlined by Fereday and Muir-Cochrane (2006). The final coding scheme appears in Table 1.

**Table 1:** Coding manual.

**Theme**		**Definition**	**Example/Quote**
Antecedents	Government Trust	One’s level of confidence in the effectiveness of governments’ efforts to manage COVID-19	Now that the lockdown restrictions have been removed, there is no need to worry. We have complete trust in the government.
	Psychological Capital	A positive psychological state that helps individuals cope with stressful situations	I think it’s okay, it’s just a matter of mentality. The government protection is actually pretty good, and you just need to have more positivity toward it.
	Past Travel Experience	One’s frequency of traveling as well as their confidence and skills associated with it	I travel a lot by myself. I want to take my senior family members on a trip, and I am confident that we can handle it.
HBM-related Variables	Perceived Susceptibility	The likelihood that one may be affected by COVID-19	Our country is very good. Jiangxi is all cleared! All cleared a long time ago!
	Perceived Severity	Perceived severity of the pandemic	There is no big risk, and the country is totally under control. It may be more dangerous in only a few places, such as Wuhan and Beijing. Other places are all safe!
	Perceived Benefits	Positive consequences of taking a trip during the pandemic	I seldom stay at home around this time of year under normal circumstances. It is so rare that we can get together. This is an opportunity, and we think we should try [traveling] regardless of the risk.
	Efficacy Beliefs	One’s confidence in protecting themselves against COVID-19 during trips	I did my research – I checked before I came. There were no cases reported in [my hometown] and [my destination]. So the places we visited are all good.
Behavioral Outcomes	Risk Reduction Behavior	One’s enactment of protective measures against COVID-19	We still have to protect ourselves. If you wear a mask and protect yourself well, the risk factor is still relatively low.

### Qualitative study findings

The qualitative study included 21 respondents (see Table A2 in Appendix). To ensure confidentiality, pseudocode was assigned to each participant for identification purposes. The sample was evenly distributed in terms of gender; nearly half of the respondents were female. Respondents were 35.4 years old on average. Most of the participants appeared to be experienced travelers: all reported traveling more than 3 times per year. When asked about travel motivations, most respondents considered their trip “*a vacation*,” “*a holiday*,” and “*a break they finally get*”—despite the lockdown having only been lifted a couple of months prior. The pandemic’s effects were evidenced by respondents’ constant engagement in self-protective measures during their trips, such as selecting safer destinations (i.e., ones in which no new cases had been reported) and transportation modes (i.e., driving their own car), wearing masks, avoiding crowded places, maintaining social distance, searching for related information, and using hand sanitizer frequently.

Additionally, respondents’ adoption of risk reduction strategies seemed to be driven by perceived benefits and self-efficacy. Perceived benefits refer to one’s belief that their actions will have positive consequences. One respondent stated, “*We need to protect ourselves at all times. If you wear a mask, it will protect you well and the risk factor will remain relatively low*.” Self-efficacy relates to respondents’ beliefs in their capacity to take protective action. This theme manifested in interviews, as most respondents indicated no difficulty complying with recommended measures. One respondent noted the importance of raising awareness of self-protection, stating that “*A lot of visitors do not do anything when no one is watching. They are not aware of the importance of protecting themselves*.”

HBM (Champion and Skinner, 2008) suggests that perceived risks affect people’s enactment of self-protective measures. In the current study, consistent with HBM, findings indicated that respondents assessed the risk level based on perceived severity and susceptibility. More specifically, tourists’ evaluations of severity were contingent on the number of reported cases in their hometown and the destination. Their perceived susceptibility was relatively low, as they generally did not believe they would contract the virus during their trips. One respondent shared, “*I do not think the pandemic is very impactful. Many regions have zero cases reported and are not very crowded. We feel pretty safe to visit all these places*.”

Besides the influences of HBM factors, our findings cast light on the effects of government trust, psychological capital, and past travel experience; all appeared to affect respondents’ decisions, travel behavior, perceived risk, and assessments of circumstances. Government trust directly influenced respondents’ risk perceptions. Despite the pandemic’s severity, most believed that their chance of contracting the virus was low, largely due to their trust in government. One respondent said, “*Now that the government has lifted travel restrictions, there is no need to worry about this. We always have complete trust in the government*.” Another interviewee elaborated on the government’s efforts to manage COVID-19: “*The policy is very strict. You must show the green code, have a temperature check, and report where you have been for the past 14 days. There is no way you will get [COVID-19] under such a strict policy*.”

Psychological capital represents “a core psychological factor of positivity in general” (Luthans et al., 2005, p. 253). Despite the overlap with HBM on self-efficacy, the qualitative results showed that psychological capital, as an aspect of positive psychology, directly influenced respondents’ sense of safety and interpretations of the situation. These patterns align with the finding that Chinese individuals’ psychological capital tends to coalesce into a general feeling (Luthans et al., 2005). A closer examination revealed slight differences in optimism and resilience. Optimism refers to a positive expectation and attitude about success in the future; such a positive attitude was common among respondents. One tourist stated, “*I am very positive about the situation. I feel safe and never worry about being infected. I have a strong heart*.” Respondents’ sense of resilience was reflected in how quickly they adapted to a changing situation, including their acceptance of the environment and compliance with government mandates.

### Hypothesis development based on qualitative findings and literature review

The findings from the qualitative study informed a conceptual framework tracing various factors’ effects on individuals’ travel behavior during the pandemic. Based on the interview findings, the HBM, and related literature, a series of hypotheses were developed to depict the relationships among the variables in the conceptual model.

#### Government trust

Trust refers to “perceived credibility and benevolence of a target of trust” (Doney and Cannon, 1997, p. 36). Trust plays a vital role in people’s behavioral decision-making under situations of risks and uncertainties (Williams and Baláž, 2021). Hence, it plays an important role in tourists’ preventive behaviors during public health crises (Zheng et al., 2022). The government could influence the public’s risk perception through governmental policies in response to and risk communication about public health crises (Yang et al., 2020). Trust in government encompasses the public’s confidence in the effectiveness of the government’s measures in dealing with public health crises and the credibility of the crisis-relevant information provided by the government (van der Weerd et al., 2011). Individuals who trust the government are also more likely to adopt the preventive measures advised by the government. Studies in public health research have empirically demonstrated that trust in government could shape the public’s risk perception, and hence can influence the public’s preventive behaviors during public health crises (van der Weerd et al., 2011; Dryhurst et al., 2020; Ye and Lyu, 2020). More specifically, our interview findings and existing studies indicated that trust in government has a positive effect on tourists’ travel behavior (whether tourists take leisure travel) and risk reduction behavior (whether tourists take preventive measures during travel) during the pandemic (Fong et al., 2020; Wong and Jensen, 2020; Zheng et al., 2022). Thus, the following hypotheses are proposed:

*H1a:* Government trust positively influences tourists’ travel behavior during the pandemic.

*H1b:* Government trust positively influences tourists’ risk reduction behavior during the pandemic.

#### Psychological capital

Psychological capital represents positive psychological resources of human beings that “go beyond human and social capital to gain a competitive advantage through investment/development of ‘who you are’” (Luthans et al., 2005, p. 253). Psychological capital consists of four dimensions, self-efficacy, hope, optimism, and resilience (Luthans et al., 2007b). Self-efficacy refers to individuals’ confidence in accomplishing a task or dealing with challenging situations (Luthans et al., 2007a). Hope reflects one’s determination to pursue goals and motivation to overcome goal-related obstacles (Snyder, 2002). Optimism captures individuals’ positive outcome expectancy (Scheier and Carver, 1985). Resilience embodies one’s ability to recover from adversity and to adapt to major life changes (Luthans et al., 2007b). Interestingly, optimism and resilience emerged in our interview findings, but hope did not appear. This is consistent with a psychological capital scale development study in China, in which hope and optimism were merged in the same construct, as Chinese people consider hope and optimism to have similar meanings (Ke et al., 2009). There is an overlap where self-efficacy was also included in HBM. Given these considerations, this study measured psychological capital through the dimensions of optimism and resilience.

Studies have suggested that optimism and resilience play critical roles when individuals cope with stressful or threatening situations (Tugade and Fredrickson, 2004; Karademas, 2006). As such, psychological capital is particularly relevant to the COVID-19 pandemic, a public health crisis that is stressful and threatening to tourists. Psychological capital could afford tourists confidence in navigating infection-related risks during travel. Tourists can then develop more positive attitudes toward the pandemic and deem the outbreak less severe and more controllable. Therefore, psychological capital could positively influence tourists’ travel behavior during the pandemic, but may negatively influence tourists’ risk reduction behavior due to their underestimated risk perceptions about the pandemic. Accordingly, the following hypotheses are proposed:

*H2a:* Psychological capital positively influences tourists’ travel behavior during the pandemic.

*H2b:* Psychological capital negatively influences tourists’ risk reduction behavior during the pandemic.

#### Past travel experience

Past travel experience, referring to the extent of travel experience that an individual accumulates in the past (Sönmez and Graefe, 1998), reflects one’s expertise and knowledge about travel. In the context of normal travel, past travel experience has been widely acknowledged to influence tourists’ future travel behavior and behavior intentions, mostly in a positive way, as past travel experience could positively influence tourists’ travel attitude, destination image perceptions, and destination familiarity (Lam and Hsu, 2006; Huang and Hsu, 2009; Liu et al., 2018). Moreover, past travel experience can affect tourist behavior through influences on travel safety and risk perceptions. It has been found that the more extensive one’s prior travel experience, the less risky they perceive regarding terrorism, health threats, food concerns, and general travel risks (Sönmez and Graefe, 1998; Lepp and Gibson, 2003; Rittichainuwat and Chakraborty, 2009; Sharifpour et al., 2014; Liu et al., 2016). During the COVID-19 pandemic, past travel experience is likely to decrease tourists’ perceived risks associated with the virus and enhance their self-efficacy in dealing with health risks during travel, thereby increasing their likelihood to travel and reducing their preventive behavior during travel. Therefore, the following hypotheses are proposed:

*H3a:* Past travel experience positively influences tourists’ travel behavior during the pandemic.

*H3b:* Past travel experience negatively influences tourists’ risk reduction behavior during the pandemic.

#### Health beliefs

HBM suggests that individuals’ health promotion and risk preventive behaviors can be illustrated by their health beliefs and risk perceptions, including one’s perceived susceptibility to a health risk, perceived severity of a health risk, perceived benefits and barriers of taking preventive measures, as well as self-efficacy in dealing with a health risk (Champion and Skinner, 2008). Perceived susceptibility refers to the perceived likelihood of being threatened by a health risk (Champion and Skinner, 2008). Perceived severity refers to the perceived seriousness of the negative outcomes of a health risk (Champion and Skinner, 2008). Perceived benefits denote the perceived benefits of taking preventive measures to reduce health risk (Champion and Skinner, 2008). Perceived barriers encompass the perceived obstacles and costs that are associated with taking preventive measures (Champion and Skinner, 2008). Self-efficacy refers to the subjective assessment of one’s own capabilities to perform preventive behaviors successfully (Champion and Skinner, 2008). According to HBM, individuals’ adoption of preventive behaviors can be positively predicted by their perceived susceptibility to and perceived severity of the health risk, perceived benefits of taking preventive measures, as well as self-efficacy, and negatively predicted by one’s perceived barriers to doing so. Perceived barriers did not appear in our interview findings and hence were not included in this study.

Government trust could enhance the public’s confidence in dealing with health crises, reduce risk perceptions, and reinforce the perceived benefits of taking preventive measures through government policies and risk communications (Fong et al., 2020; Wong and Jensen, 2020; Yang et al., 2020). Powered by positive psychological states like optimism and resilience, health belief-related variables can foster tourists’ confidence in dealing with COVID-19 infection risk during travel, reduce their risk perception, and even results in a positive attitude toward tourism and travel during the pandemic.

Similarly, past travel experience could also mitigate risk perceptions and enhance tourists’ self-efficacy in dealing with health risks during travel. This is mainly because past travel experience can evoke a sense of familiarity, which is associated with feelings of safety and assurance (Liu et al., 2016; Tan and Wu, 2016), and it can enhance tourists’ confidence through greater destination knowledge and travel expertise, mitigating their risk perceptions and promoting their travel intentions (Tan and Wu, 2016).

Based on the above discussion, health bliefs, including perceived susceptibility, perceived severity, perceived benefits and self-efficacy, could serve as the underlying mechanisms in the relationships between government trust, psychological capital, past travel experiences and health risk preventive behaviors (including travel behavior and risk reduction behavior). Hence, the following hypotheses are proposed:

*H4a:* Health beliefs mediate the relationship between government trust and travel behavior during the pandemic.

*H4b:* Health beliefs mediate the relationship between government trust and risk reduction behavior during the pandemic.

*H5a:* Health beliefs mediate the relationship between psychological capital and travel behavior during the pandemic.

*H5b:* Health beliefs mediate the relationship between psychological capital and risk reduction behavior during the pandemic.

*H6a:* Health beliefs mediate the relationship between past travel experience and travel behavior during the pandemic.

*H6b:* Health beliefs mediate the relationship between past travel experience and risk reduction behavior during the pandemic.

**Figure 2:**
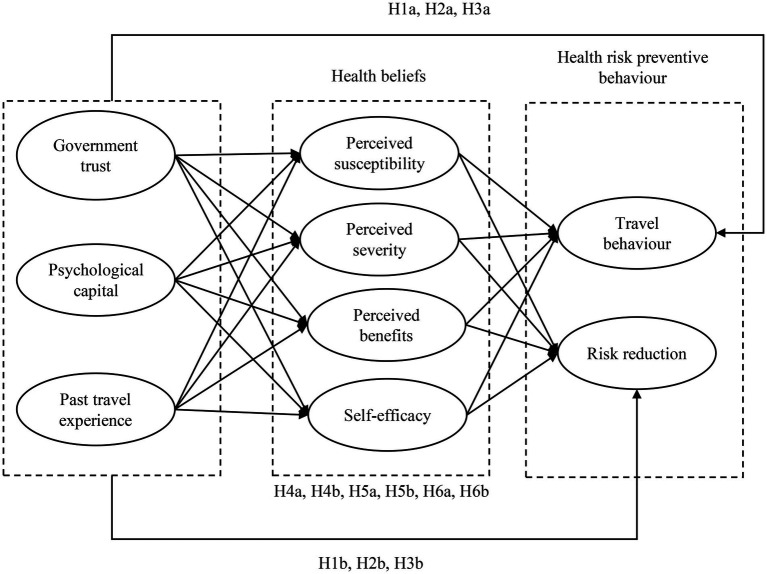
Conceptual model.

Our conceptual model and hypotheses are presented in Figure 2.

## Quantitative study

### Quantitative research design

#### Measurement

The quantitative phase was conducted to validate qualitative findings, test the hypotheses and to generalize results to a larger population. A questionnaire was developed based on the qualitative interviews and relevant literature. The questionnaire included items related to (1) people’s travel behavior during the pandemic; (2) their perceived susceptibility, perceived severity, perceived benefits, and efficacy beliefs related to traveling and COVID-19; (3) their intentions to engage in risk reduction behavior when traveling during the pandemic; (4) government trust; (5) psychological capital; and (6) past travel experience and other demographic variables. All items were measured using a 7-point Likert scale (1 = “strongly disagree,” 7 = “strongly agree”); see Table A1 in Appendix for scale items and sources.

#### Data collection

The questionnaire was drafted in English and translated into Chinese. A back-translation method was used to ensure the accuracy and equivalence of translation. The questionnaire was then pilot tested with 127 individuals in July and August 2020. The reliability coefficients of the measurement scales ranged from 0.68 to 0.91. Based on the analysis results and participant feedback, the questionnaire was slightly revised to make it easier to understand and more user-friendly. The final data were gathered in August 2020 through a cross section of respondents sampled in seven major (Tier 1 and Tier 2) cities in China: Beijing, Shanghai, Guangzhou, Shenzhen, Chengdu, Chongqing, and Wuhan were selected from the top 10 cities with the highest tourism consumption in 2019 (Ctrip, 2019). Seven research assistants were hired, trained, and sent to these cities to collect data. The survey was hosted on the Qualtrics website, and a unique QR code was generated for each city. A non-random sampling method (i.e., intercepting every third passer-by) was used to select survey participants in crowded locations (e.g., residential areas, shopping malls, parks). Respondents who completed the survey received a small gift. The questionnaire took about 12 minutes to complete on average. A total of 1,239 responses comprised the initial dataset.

#### Data analysis

SPSS 27.0 and R (4.0.3) software were used to analyze the data. First, a screening process was conducted to exclude incomplete responses and those with completion time exceeding 24 h, resulting in a final sample of 901 usable responses. Second, descriptive analyses were carried out in SPSS to profile the respondents. Third, the measurement model’s reliability, validity, and common method bias were checked using R. Finally, the structural model was tested and estimated in R with weighted least squares mean- and variance-adjusted (WLSMV) estimation.

The WLSMV estimator was used in SEM for two reasons. First, the research model included a binary outcome variable, travel behavior. Second, Mardia’s test was performed to check the multivariate normality of data; results showed that the data did not follow a multivariate normal distribution (Mardia Skewness = 185.01, *p* < 0.001; Mardia Kurtosis = 1655.86, *p* < 0.001). WLSMV estimation is appropriate for analyzing categorical data, including binary/ordinal variables and non-normal data (McKay and Andretta, 2017; Huang et al., 2020).

### Quantitative study findings

A total of 901 responses were included in the final sample, and respondents’ demographic profiles can be found in Table A3 in Appendix. The sample included slightly more male than female respondents (53% vs. 47%). Respondents above age 20 were roughly evenly distributed across age groups. Most were married (68%). Many respondents had completed either college (30%) or high school/vocational high school (26%). Slightly less than half of the respondents were employed full- or part-time (40%), followed by self-employed individuals (21%). Nearly two-thirds of respondents (64%) earned a monthly household income of 4,000–19,999 RMB (approximately US$608–$3,044). This sample was representative of Chinese urban citizens in terms of gender, age, and marital status according to the 2019 Population Sample Survey (National Bureau of Statistics of China, 2019).

#### Measurement model

Structural equation modeling (SEM) with the WLSMV estimator was employed to analyze the quantitative data. The results indicated a good model fit (chi-square = 1,279.647, *df* = 434, SRMR = 0.035, CFI = 0.979, TLI = 0.976, RMSEA = 0.047). Reliability was evaluated using Cronbach’s coefficient alpha, composite reliability, indicator reliability (i.e., squared indicator loading), and average variance extracted (AVE). The constructs demonstrated sound reliability (Table 2): all Cronbach’s alpha coefficients and composite reliability coefficients exceeded 0.8, all indicator reliability values were larger than 0.5, and all AVE values were greater than 0.6 (Hair et al., 2019). The significant item loadings and high AVE values (>0.6) showed that all constructs possessed good convergent validity. Additionally, all squared correlations were smaller than the AVE of each construct, and all heterotrait–monotrait ratios were smaller than 0.85 (Table 3), supporting the constructs’ good discriminant validity (Henseler et al., 2015). Common method bias was checked using Harman’s single-factor test (Podsakoff et al., 2003) and was not a major concern.

**Table 2:** Measurement properties.

Scale items	Cronbach’s *α*	Composite reliability	AVE	Factor loading	Mean (SD)
Perceived susceptibility (SUSCEP)	0.863	0.879	0.710		
SUSCEP1				0.875	3.63 (1.683)
SUSCEP2				0.903	3.82 (1.657)
SUSCEP3				0.740	3.50 (1.734)
Perceived severity (SEVE)	0.894	0.927	0.761		
SEVE1				0.885	5.60 (1.459)
SEVE2				0.881	5.65 (1.373)
SEVE3				0.911	5.79 (1.306)
SEVE4				0.809	5.83 (1.384)
Perceived benefits (BENE)	0.863	0.902	0.754		
BENE1				0.875	5.59 (1.217)
BENE2				0.864	5.78 (1.122)
BENE3				0.866	5.72 (1.145)
Self-efficacy (EFFIC)	0.832	0.854	0.663		
EFFIC1				0.894	4.84 (1.385)
EFFIC2				0.719	4.15 (1.490)
EFFIC3				0.820	4.54 (1.461)
Government trust (GOV)	0.908	0.941	0.798		
GOV1				0.853	5.96 (1.113)
GOV2				0.910	6.05 (1.036)
GOV3				0.896	6.10 (1.026)
GOV4				0.914	6.05 (1.076)
Optimism (OPT)	0.845	0.875	0.636		
OPT1				0.811	5.22 (1.328)
OPT2				0.865	5.19 (1.274)
OPT3				0.731	4.72 (1.465)
OPT4				0.778	5.36 (1.311)
Resilience (RESIL)	0.919	0.933	0.701		
RESIL1				0.838	5.09 (1.325)
RESIL2				0.848	4.95 (1.379)
RESIL3				0.819	4.99 (1.344)
RESIL4				0.876	5.15 (1.269)
RESIL5				0.788	5.11 (1.275)
RESIL6				0.851	4.98 (1.344)
Psychological capital (PSY, second order construct)	-	-	-		
Optimism				0.969	–
Resilience				0.981	–
Past travel experience (EXPE)	0.810	0.857	0.753		
EXPE1				0.736	3.60 (1.755)
EXPE2				0.982	3.22 (1.593)
Risk reduction (RR)	0.897	0.928	0.810		
RR1				0.863	6.04 (1.278)
RR2				0.933	6.13 (1.146)
RR3				0.903	6.06 (1.261)
Travel behavior (BEHAV)	Frequency: no = 554, yes = 347				

**Table 3:** Correlations and heterotrait–monotrait ratios.

	**GOV**	**PSY**	**EXPE**	**SUSCEP**	**SEVE**	**BENE**	**EFFIC**	**RR**
GOV	1.000	0.486	0.070	0.064	0.452	0.505	0.220	0.505
PSY	0.468	1.000	0.133	0.225	0.316	0.371	0.385	0.237
EXPE	0.072	0.142	1.000	0.174	0.075	0.086	0.265	0.085
SUSCEP	0.044	0.211	−0.168	1.000	0.313	0.070	0.073	0.057
SEVE	0.451	0.300	−0.024	0.311	1.000	0.501	0.137	0.577
BENE	0.506	0.359	0.086	0.066	0.501	1.000	0.316	0.590
EFFIC	0.232	0.395	0.256	−0.014	0.142	0.332	1.000	0.115
RR	0.504	0.222	0.068	0.003	0.575	0.587	0.113	1.000

Correlations are presented in the lower triangle of the matrix; heterotrait–monotrait ratios are presented in the upper triangle of the matrix.

#### Structural model

**Figure 3:**
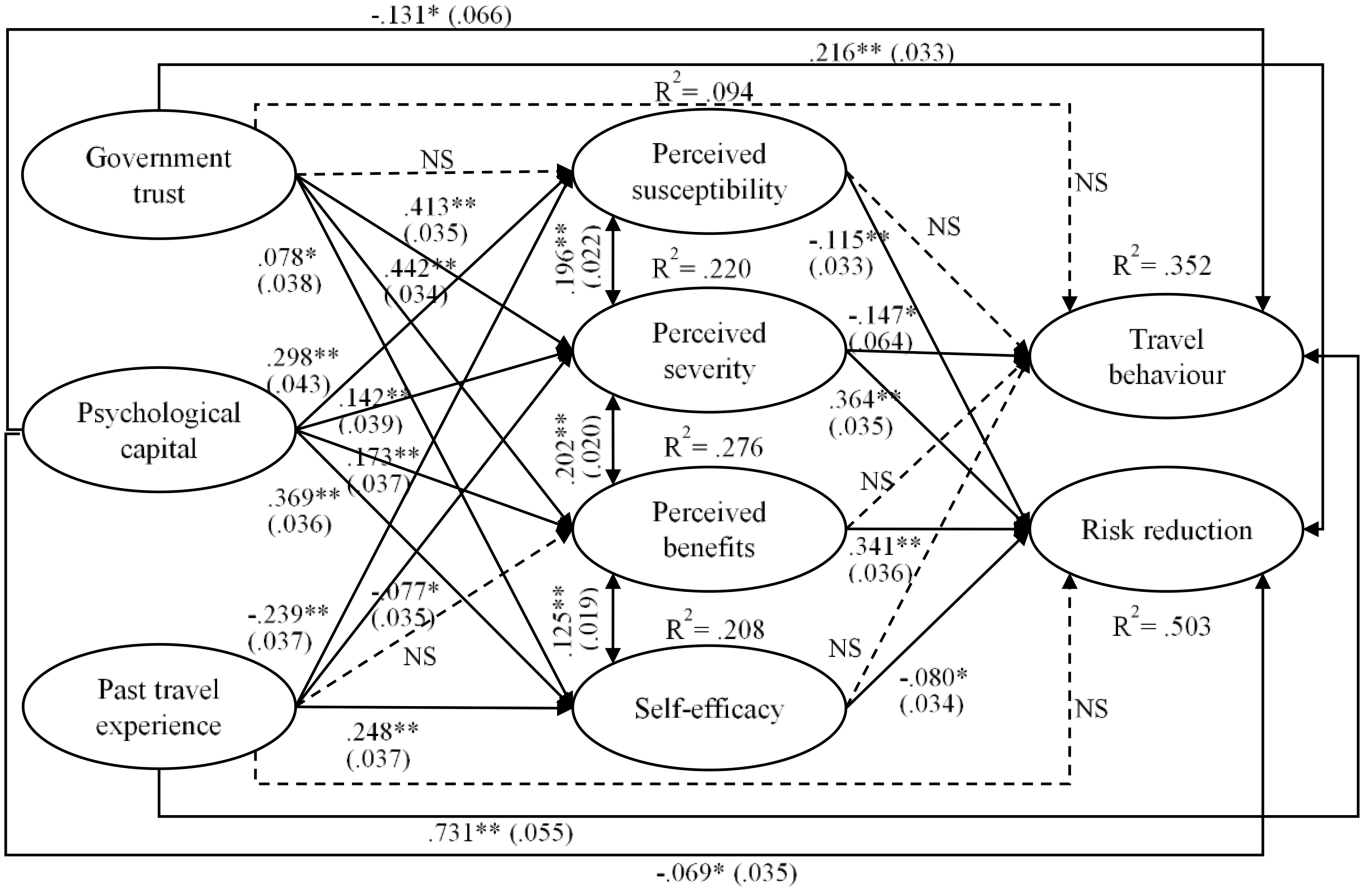
Results of structural model. Values in parentheses are standard errors; ^*^ significant at 0.05; ^**^ significant at 0.01.

The model demonstrated a good fit (chi-square =  2,132.426, *df* = 464, SRMR = 0.050, CFI = 0.960, TLI = 0.954, RMSEA = 0.063). The correlation analysis and modification index showed that perceived susceptibility and perceived benefits were each correlated with perceived severity, while self-efficacy was correlated with perceived benefits. Correlation paths were hence added among these constructs. The changes significantly improved the structural model fit (chi-square = 1264.517, *df* = 461, SRMR = 0.036, CFI = 0.981, TLI = 0.978, RMSEA = 0.044). Estimated results appear in Figure 3. Trust in government, psychological capital, and past travel experience collectively explained 9.4% of the variance in perceived susceptibility, 22% of the variance in perceived severity, 27.6% of the variance in perceived benefits, and 20.8% of the variance in self-efficacy. The model further explained 35.2% of the variance in travel behavior and 50.3% of the variance in risk reduction behavioral intention.

In terms of path coefficients, trust in government had significantly positive effects on perceived severity (*β* = 0.413, *p* < 0.01), perceived benefits (*β* = 0.442, *p* < 0.01), and self-efficacy (*β* = 0.078, *p* < 0.05) but no significant impact on perceived susceptibility. Psychological capital exerted significantly positive effects on all health belief–related constructs (*β*_SUSCEP_ = 0.298, *p* < 0.01; *β*_SEVE_ = 0.142, *p* < 0.01; *β*_BENE_ = 0.173, *p* < 0.01; *β*_EFFIC_ = 0.369, *p* < 0.01). Past travel experience had significantly negative impacts on perceived susceptibility (*β* = −0.239, *p* < 0.01) and severity (*β* = −0.077, *p* < 0.05). This element also had a significantly positive effect on self-efficacy (*β* = 0.248, *p* < 0.01) but no significant impact on perceived benefits. Further, travel behavior was negatively influenced by perceived susceptibility (*β* = −0.147, *p* < 0.01) and psychological capital (*β* = −0.131, *p* < 0.01) but was strongly positively affected by past travel experience (*β* = 0.731, *p* < 0.01). Risk reduction was adversely affected by perceived susceptibility (*β* = −0.115, *p* < 0.01), self-efficacy (*β* = −0.080, *p* < 0.05), and psychological capital (*β* = −0.069, *p* < 0.05); it was positively influenced by perceived severity (*β* = 0.364, *p* < 0.01), perceived benefits (*β* = 0.341, *p* < 0.01), and trust in government (*β* = 0.216, *p* < 0.01).

The indirect effects of trust in government, psychological capital, and past travel experience on travel behavior and risk reduction through health beliefs were tested *via* a bias-corrected bootstrapping procedure with 10,000 samples. Trust in government had a significant indirect effect on travel behavior through the mediation of perceived severity [*β* = −0.061, SE = 0.028, *p* < 0.05, 95% confidence interval (CI): [−0.121, −0.010)] and a significant indirect effect on risk reduction through the mediation of perceived severity [*β* = 0.150, SE = 0.026, *p* < 0.01, 95% CI: (0.105, 0.207)] and perceived benefits [*β* = 0.151, SE = 0.026, *p* < 0.01, 95% CI: (0.104, 0.207)]. Psychological capital exerted a significant indirect impact on risk reduction through the mediation of perceived susceptibility [*β* = −0.034, SE = 0.013, *p* < 0.05, 95% CI: (−0.065, −0.012)], perceived severity [*β* = 0.052, SE = 0.021, *p* < 0.05, 95% CI: (0.014, 0.095)], and perceived benefits [*β* = 0.059, SE = 0.020, *p* < 0.01, 95% CI: (0.024, 0.103)]. Past travel experience had a significant indirect effect on risk reduction through the mediation of perceived susceptibility [*β* = 0.028, SE = 0.011, *p* < 0.05, 95% CI: (0.010, 0.056)]. Neither psychological capital nor past travel experience had significant indirect effects on travel behavior through the mediation of health beliefs.

The hypotheses testing results are summarized in Table 4.

**Table 4:** Summary of hypotheses testing results.


**Hypohteses**		**Results**
H1a	Government trust positively influences tourists’ travel behavior during the pandemic	Unsupported
H1b	Government trust positively influences tourists’ risk reduction behavior during the pandemic	Supported
H2a	Psycholoical capital positively influences tourists’ travel behavior during the pandemic	Unsupported
H2b	Psycholocal capital negatively influences tourists’ risk reduction behavior during the pandemic	Supported
H3a	Past travel experience positively influences tourists’ travel behavior during the pandemic	Supported
H3b	Past travel experience negatively influences tourists’ risk reduction behavior during the pandemic	Unsupported
H4a	Health beliefs mediate the relationship between government trust and travel behavior during the pandemic	Partly supported
H4b	Health beliefs mediate the relationship between government trust and risk reduction behavior during the pandemic	Partly supported
H5a	Health beliefs mediate the relationship between psychological capital and travel behavior during the pandemic	Unsupported
H5b	Health beliefs mediate the relationship between psychological capital and risk reduction behavior during the pandemic	Partly supported
H6a	Health beliefs mediate the relationship between past travel experience and travel behavior during the pandemic	Unsupported
H6b	Health beliefs mediate the relationship between past travel experience and risk reduction behavior during the pandemic	Partly supported

## Discussion and conclusion

Travel during the pandemic is essential to destination recovery, especially considering the complexity and unpredictability of the current situation. This study explored travelers’ health beliefs and behaviors during a pandemic through a mixed-methods research design. The quantitative and qualitative components of this research are complementary. The qualitative phase substantiated HBM’s applicability to Chinese tourists’ actual travel behavior and tendency to reduce risk. This phase also elucidated the roles of government trust, psychological capital, and past travel experience as antecedents. The quantitative phase further validated the qualitative findings, confirming relationships among all these key constructs.

Consistent with earlier observations (Wang et al., 2019), tourists tend to adopt various risk reduction strategies to avert potential threats. Different from studies on tourists’ travel-related behavioral intentions during and after the pandemic (Liu-Lastres et al., 2021; Neuburger and Egger, 2020; Zheng et al., 2021), our work indicated that the Chinese public acknowledged the risks associated with COVID-19 but did not necessarily avoid traveling, especially among experienced travelers. Instead, nearly 40% of respondents in the quantitative study reported having taken leisure trips during the pandemic. As reflected by the qualitative study findings, the pandemic did not significantly alter individuals’ perspectives on vacationing. The quantitative and qualitative findings both indicated that respondents’ willingness to travel during this time was primarily rooted in accumulated past travel experience and a lower level of perceived severity resulting from government trust.

Additionally, this study adopted HBM to understand individuals’ travel behavior and intentions to adopt risk reduction strategies. As expected, the model was more effective in explaining respondents’ propensity to engage in risk reduction than their actual travel behavior. The qualitative findings offer a possible explanation for these discrepancies, such that people might not automatically avoid traveling as a self-protective measure. Instead, wearing masks and social distancing were common risk reduction strategies. The quantitative results pointed to a positive association between perceived benefits and tourists’ intentions to engage in self-protective measures.

Two core variables of HBM, self-efficacy and perceived susceptibility, are normally positively related to one’s engagement in protective travel behavior (Wang et al., 2019). Interestingly, the quantitative study showed both self-efficacy and perceived susceptibility are negatively related to the sample’s likelihood of adopting risk reduction strategies. The qualitative findings provide further insight into this outcome: some tourists possessed so much trust and confidence in the government that they did not believe they urgently needed to engage in self-protection. Zheng et al. (2022) pointed out that government trust can reduce travel fear and increase travel avoidance. Our study adds to this stream of literature, indicating that government trust can be a double-edged sword such that people may feel empowered to take trips without adhering to recommended protective measures.

Lastly, moving beyond the scope of HBM, the qualitative study uncovered three antecedents influencing people’s health beliefs and behavior during the pandemic: (1) government trust, which we discussed above; (2) psychological capital; and (3) past travel experience. Our quantitative results showed that psychological capital was related to all four HBM variables but also affected respondents’ intentions to engage in risk reduction. These findings suggest that three HBM variables (i.e., perceived severity, perceived susceptibility, and perceived benefits) mediate the relationship between psychological capital and individuals’ intentions to engage in risk reduction strategies. In other words, individuals possessing greater psychological capital appear more likely to enact self-protective measures due to a lower level of perceived susceptibility, a higher level of perceived severity, and a higher level of perceived benefits. These patterns are congruent with our qualitative findings, which demonstrated that tourists exhibiting a stronger mentality were more apt to accept the present reality, acknowledge the pandemic’s severity, and take protective action while enjoying their trips. Additionally, the findings showed that experienced travelers were more apt to adopt risk reduction strategies due to the effects of perceived susceptibility and severity. This trend echoes earlier research (Sharifpour et al., 2014; Wang et al., 2019) demonstrating that experienced travelers tend to be confident, knowledgeable, and make informed decisions regardless of the level of perceived threat.

The key findings of this study also offer several practical implications. First, the COVID-19 outbreak, which is a unique case, is a global pandemic and a veritable public health risk. Accordingly, government initiatives have become essential to managing this crisis. As our findings show, effective government efforts can help control the outbreak, reassure the public, increase individuals’ trust in the government, affect their judgment of the situation, and afford them confidence in taking leisure trips. Therefore, to accelerate destinations’ recovery from the pandemic, government efforts should be transparent and well communicated to the public. Disseminating consistent, timely, and proper messages to society should be prioritized in the government’s response effort, which is essential in enhancing individuals’ government trust. Also, broadcasting these messages to different regions through various channels (e.g., TV, radio, website) is critical so that every member of society can access these key messages.

Second, our qualitative findings suggest that people have assessed situational severity based on the number of reported COVID-19 cases and rely heavily on government guidance. Therefore, destination marketing and managerial messaging should highlight such information. Campaigns can focus on the government’s efforts in dealing with the pandemic, local government’s measures to assure tourists’ safety, as well as preventive measures recommended by the government. Addressing the authority aspect of the information should always be featured. Following this line of discussion, a collaborative approach to crisis management seems feasible under these circumstances. A variety of groups, ranging from the government and scientists to private enterprises, should be included in public health initiatives (e.g., the COVID-19 outbreak). The tourism and travel industry, destination management organizations and industry associations for example, should take an active role in this collaboration.

Third, our findings suggest that people do not always consider travel avoidance a risk reduction strategy. Therefore, a positive association between the tourism and travel industry and crisis responses can create positive publicity. In light of this, major players in the tourism and travel industry should demonstrate their corporate social responsibility endeavors and contribute to the crisis response efforts. Examples include donations, special discounts for medical personnel, and participation in disaster relief efforts. These measures can improve their reputation and public image as well as reinforce the distinction between travel and self-protection to attract tourists even in times of crisis.

Fourth, we found that travel experience plays a key role in people’s health beliefs and behavior. This notion illuminates a primary segment for destination recovery marketing, namely repeat tourists. Domestic tourism is a typical key segment for destination recovery marketing. Similarly, destinations should extend their efforts to encourage repeat visits. New promotions such as special events and discounts will all be beneficial in attracting previous visitors to revisit and/or enhancing their destination loyalty. Additionally, given the importance of psychological capital, it is imperative that destinations and tourism businesses monitor the market closely and attend to tourists’ overall perceptions, attitudes, and sentiments. Psychographic segmentation on the basis of individuals’ overall attitudes toward the pandemic—and travel in general—can inform effective destination recovery strategies. Thus, market reports and sentimental analyses have been imperative for the industry to evaluate the market and develop research- and evidence-based marketing recovery strategies.

Lastly, this research is subject to several limitations that leave room for future studies. First, we referred only to Chinese tourists; hence, the generalizability of our findings is limited. Scholars could test our model with individuals from other countries, as risk perceptions vary by culture (Weber and Hsee, 1998), and people hold different attitudes toward the government in Eastern and Western regions (Yang et al., 2020). Second, our findings were based on Chinese residents’ domestic travel, as outbound leisure travel was not permitted during the data collection period. Subsequent work could explore factors predicting outbound travel behavior during a pandemic once such travel is allowed. Third, this study was guided by HBM. Other theoretical models, such as protection motivation theory, can be adopted to explore similar issues and to compare with our conclusions.

## Data availability statement

The raw data supporting the conclusions of this article are available upon request.

## Ethics statement

The studies involving human participants were reviewed and approved by Jiangxi Normal University. The patients/participants provided their written informed consent to participate in this study.

## Author contributions

HL: conceptualization, quantitative research design, quantitative data analysis, and writing the manuscript. BL-L: conceptualization, quantitative research design, and writing the manuscript. LZ: conceptualization, qualitative research design and data collection and analysis, quantitative data collection, and writing the manuscript. HD: conceptualization and writing the manuscript. All authors contributed to the article and approved the submitted version.

## Funding

This work was supported by the National Natural Science Foundation of China under grant no. 41961026 (to LZ). This work was also supported by the Library Open Access Fund from University of Surrey.

## Conflict of interest

The authors declare that the research was conducted in the absence of any commercial or financial relationships that could be construed as a potential conflict of interest.

## Publisher’s note

All claims expressed in this article are solely those of the authors and do not necessarily represent those of their affiliated organizations, or those of the publisher, the editors and the reviewers. Any product that may be evaluated in this article, or claim that may be made by its manufacturer, is not guaranteed or endorsed by the publisher.

## Supplementary material

The Supplementary material for this article can be found online at: https://www.frontiersin.org/articles/10.3389/fpsyg.2022.1015421/full#supplementary-material
