# Supplementary material for: Travel despite the COVID-19 pandemic: Implications for tourism recovery
Source: Front Psychol. 2022 Oct 5;13:1015421. doi: 10.3389/fpsyg.2022.1015421 (PMC9580466; doi:10.3389/fpsyg.2022.1015421)
Supplement: Supplementary file 1 [file Table_1.docx]

**Appendix**

**Table A1 Measurement Items and Sources.**

| Scale items | Sources |
| --- | --- |
| Perceived susceptibility (SUSCEP) |  |
| 1. My chances for getting COVID-19 during the trip are high. | Witte (1996) |
| 1. It is very likely that I will get COVID-19 during the trip. |  |
| 1. In general, I am very susceptible to infectious diseases, like cold and flu, as well as COVID-19. |  |
|  |  |
| Perceived severity (SEVE) | Witte (1996) |
| 1. If I were to get COVID-19 during the trip, I would experience serious negative consequences. |  |
| 1. If I were to get COVID-19 during the trip, it would have a severe negative impact on my health. |  |
| 1. If I were to get COVID-19 during the trip, it would be harmful to my well-being. |  |
| 1. If I were to get COVID-19 during the trip, my whole trip would be craped. |  |
|  |  |
| Perceived benefits (BENE) | Wang, Liu-Lastres, Ritchie and Mills (2019) |
| 1. Taking preventive measures such as social distancing will prevent me from getting infected with Coronavirus during the trip. |  |
| 1. Taking preventive measures such as social distancing is beneficial to my health during the trip. |  |
| 1. Taking preventive measures such as social distancing will help reduce health risks during the trip. |  |
|  |  |
| Self-efficacy (EFFIC) | Witte (1996) |
| 1. I am able to prevent getting COVID-19 infections during my travel |  |
| 1. I can easily prevent getting COVID-19 infections during the trip |  |
| 1. I have the skills to deal with COVID-19 infections during the trip |  |
|  |  |
| Government trust (GOV) | van der Weerd et al. (2011) |
| 1. How much trust do you generally have in information provided by the government about the COVID-19? |  |
| 1. How much trust do you have in the measures taken by the government against the COVID-19 pandemic? |  |
| 1. How much trust do you have in the government with respect to fighting the COVID-19 pandemic? |  |
| 1. How much trust do you have in the decisiveness of the government in taking safety measures against the COVID-19 pandemic? |  |
|  |  |
| Psychological capital (PSY, second order construct) |  |
| Optimism | Ke, Sun and Li (2009) |
| 1. I'm happy about everything, almost every day. |  |
| 1. I can get over myself quickly after something unpleasant has happened. |  |
| 1. I feel optimistic and almost never get depressed. |  |
| 1. In uncertain times, I usually expect the best. |  |
|  |  |
| Resilience | Smith et al. (2008) |
| 1. I tend to bounce back quickly after hard times. |  |
| 1. I tend to easily get through stressful events. |  |
| 1. It does not take me long to recover from a stressful event. |  |
| 1. It is easy for me to snap back when something bad happens. |  |
| 1. I usually come through difficult times with little trouble. |  |
| 1. It does not take me long to get over set-backs in my life. |  |
|  |  |
| Past travel experience (EXPE) | Sönmez and Sirakaya (2002); Beerli and Martı́n (2004) |
| 1. What is your overall level of travel experience? (1 = no experience at all, 7 = a lot of travel experience) |  |
| 1. In general (before the outbreak of COVID-19), what is your general travel frequency (including travel for any purposes, 1 = very infrequently, 7 = very frequently) |  |
|  |  |
| Risk reduction (RR) | Interview findings |
| 1. I would want to search more information about COVID-19 related to the destination/attractions before and during the trip. |  |
| 1. I would like to participate in behaviors that protect myself against COVID-19 during the trip. |  |
| 1. I would follow the guidelines at the destination/attractions, such as keep safe distance with others, in order to protect myself against COVID-19 infection. |  |
|  |  |
| Travel behavior (BEHAV) | - |
| Since the lift of national lockdown in China (since May 2020), how many times did you travel for leisure purpose (including domestic and outbound travel)? (dummy coded, 0 = No, 1 = Yes) |  |

**References:**

Beerli A and Martı́n JD (2004) Tourists’ characteristics and the perceived image of tourist destinations: A quantitative analysis—a case study of Lanzarote, Spain. *Tourism Management* 25(5): 623-636. DOI: 10.1016/j.tourman.2003.06.004.

Ke J, Sun J and Li Y (2009) Psychological capital: Chinese indigenous scale’s development and its validity comparison with the western scale. *Acta Psychologica Sinica* 41(9): 875-888. DOI: 10.3724/SP.J.1041.2009.00875.

Smith BW, Dalen J, Wiggins K, Tooley E, Christopher P and Bernard J (2008) The brief resilience scale: Assessing the ability to bounce back. *International Journal of Behavioral Medicine* 15(3): 194-200. DOI: 10.1080/10705500802222972.

Sönmez S and Sirakaya E (2002) A distorted destination image? The case of Turkey. *Journal of Travel Research* 41(2): 185-196. DOI: 10.1177/004728702237418.

van der Weerd W, Timmermans DR, Beaujean DJ, Oudhoff, J and Van Steenbergen, JE (2011) Monitoring the level of government trust, risk perception and intention of the general public to adopt protective measures during the influenza A (H1N1) pandemic in the Netherlands. *BMC Public Health* 11(1): 1-12. DOI: 10.1186/1471-2458-11-575.

Wang J, Liu-Lastres B, Ritchie BW and Mills DJ (2019) Travellers' self-protections against health risks: An application of the full Protection Motivation Theory. *Annals of Tourism Research* 78:102743. DOI: 10.1016/j.annals.2019.102743.

Witte K (1996) Predicting risk behaviors: Development and validation of a diagnostic scale. *Journal of Health Communication* 1(3): 317-342. DOI: 10.1080/108107396127988.
