# Supplementary material for: Travel despite the COVID-19 pandemic: Implications for tourism recovery
Source: Front Psychol. 2022 Oct 5;13:1015421. doi: 10.3389/fpsyg.2022.1015421 (PMC9580466; doi:10.3389/fpsyg.2022.1015421)
Supplement: Supplementary file 2 [file Table_2.docx]

**Appendix**

**Table A2. Participants' Profiles in the Qualitative Study.**

| **ID** | **Age** | **Gender** | **Format** | **Residency** | **Travel Frequency** |
| --- | --- | --- | --- | --- | --- |
| ID1 | 30 | M | site interview | Beijing | More than 3 times a year |
| ID2 | 30 | F | site interview | Hunan | More than 7 times a year |
| ID3 | 28 | F | site interview | Guangdong | More than 5 times a year |
| ID4 | 25 | M | site interview | Shanxi | More than 3 times a year |
| ID5 | 26 | F | phone interview | Jiangxi | More than 7 times a year |
| ID6 | 21 | M | site interview | Jiangxi | More than 3 times a year |
| ID7 | 26 | F | phone interview | Shanghai | More than 3 times a year |
| ID8 | 26 | M | phone interview | Guangdong | More than 10 times a year |
| ID9 | 33 | M | phone interview | Jiangxi | More than 5 times a year |
| ID10 | 34 | M | phone interview | Jiangxi | More than 5 times a year |
| ID11 | 42 | M | phone interview | Jiangxi | More than 20 times a year |
| ID12 | 65 | F | site interview | Guangdong | More than 6 times a year |
| ID13 | 25 | F | site interview | Guangdong | More than 13 times a year |
| ID14 | 73 | M | site interview | Jiangxi | More than 4 times a year |
| ID15 | 30 | F | site interview | Jiangxi | More than 8 times a year |
| ID16 | 50 | F | site interview | Jiangxi | More than 7 times a year |
| ID17 | 50 | M | site interview | Jiangxi | More than 7 times a year |
| ID18 | 45 | F | site interview | Jiangxi | More than 3 times a year |
| ID19 | 20 | M | site interview | Jiangxi | More than 8 times a year |
| ID20 | 43 | F | site interview | Fujian | More than 3 times a year |
| ID21 | 22 | M | site interview | Jiangxi | More than 3 times a year |
