# Supplementary material for: Travel despite the COVID-19 pandemic: Implications for tourism recovery
Source: Front Psychol. 2022 Oct 5;13:1015421. doi: 10.3389/fpsyg.2022.1015421 (PMC9580466; doi:10.3389/fpsyg.2022.1015421)
Supplement: Supplementary file 3 [file Table_3.docx]

**Appendix**

**Table A3. Sample Profiles in the Quantitative Study.**

| **Demographic** | **Category** | **Frequency** | **Percentage** |
| --- | --- | --- | --- |
| Gender | Male | 474 | 52.7% |
|  | Female | 425 | 47.3% |
|  | Total | 899 | 100.0% |
| Age | 18–19 | 20 | 2.2% |
|  | 20–29 | 176 | 19.5% |
|  | 30–39 | 194 | 21.5% |
|  | 40–49 | 193 | 21.4% |
|  | 50–59 | 166 | 18.4% |
|  | 60 or above | 152 | 16.9% |
|  | Total | 901 | 100.0% |
| Marital status | Single/Never married | 220 | 24.5% |
|  | Married | 615 | 68.4% |
|  | Separated/Divorced/Widowed | 59 | 6.6% |
|  | Others | 5 | 0.6% |
|  | Total | 899 | 100% |
| Education | Middle school or less | 182 | 20.2% |
|  | High school/vocational high school | 235 | 26.1% |
|  | Some college | 157 | 17.5% |
|  | College graduate | 269 | 29.9% |
|  | Graduate work/advanced degree | 56 | 6.2% |
|  | Total | 899 | 100.0% |
| Employment | Employed full time | 318 | 35.3% |
|  | Employed part time | 41 | 4.6% |
|  | Self-employed | 184 | 20.5% |
|  | Housewife/homemaker | 49 | 5.5% |
|  | Temporarily unemployed/looking for work | 25 | 2.8% |
|  | Retired | 158 | 17.6% |
|  | Student | 69 | 7.7% |
|  | Other | 55 | 6.1% |
|  | Total | 899 | 100% |
| Monthly household income | Less than RMB 4,000 | 98 | 10.9% |
|  | RMB 4,000 to 6,999 | 234 | 26% |
|  | RMB 7,000 to 9,999 | 146 | 16.2% |
|  | RMB 10,000 to 19,999 | 194 | 21.5% |
|  | RMB 20,000 to 29,999 | 71 | 7.9% |
|  | RMB 30,000 to 39,999 | 28 | 3.1% |
|  | RMB 40,000 to 49,999 | 16 | 1.8% |
|  | RMB 50,000 or above | 22 | 2.4% |
|  | Prefer not to answer | 90 | 10% |
|  | Total | 899 | 100.0% |
| City | Beijing | 140 | 15.5% |
|  | Shenzhen | 122 | 13.5% |
|  | Shanghai | 121 | 13.4% |
|  | Guangzhou | 129 | 14.3% |
|  | Wuhan | 131 | 14.5% |
|  | Chengdu | 129 | 14.3% |
|  | Chongqing | 129 | 14.3% |
|  | Total | 901 | 100.0% |
